# Fast Synaptically Activated Calcium and Sodium Kinetics in Hippocampal Pyramidal Neuron Dendritic Spines

**DOI:** 10.1523/ENEURO.0396-22.2022

**Published:** 2022-11-25

**Authors:** Kenichi Miyazaki, William N. Ross

**Affiliations:** Department of Physiology, New York Medical College, Valhalla, NY 10595

**Keywords:** AMPA, calcium, NMDA, sodium, spine

## Abstract

An accurate assessment of the time course, components, and magnitude of postsynaptic currents is important for a quantitative understanding of synaptic integration and signaling in dendritic spines. These parameters have been studied in some detail in previous experiments, primarily using two-photon imaging of [Ca^2+^]_i_ changes and two-photon uncaging of glutamate. However, even with these revolutionary techniques, there are some missing pieces in our current understanding, particularly related to the time courses of synaptically evoked [Ca^2+^]_i_ and [Na^+^]_i_ changes. In new experiments, we used low-affinity, linear Na^+^ and Ca^2+^ indicators, laser fluorescence stimulation, and a sensitive camera-based detection system, combined with electrical stimulation and two-photon glutamate uncaging, to extend measurements of these spine parameters. We found that (1) almost all synaptically activated Na^+^ currents in CA1 hippocampal pyramidal neuron spines in slices from mice of either sex are through AMPA receptors with little Na^+^ entry through voltage-gated sodium channels (VGSCs) or NMDA receptor channels; (2) a spectrum of sodium transient decay times was observed, suggesting a spectrum of spine neck resistances, even on the same dendrite; (3) synaptically activated [Ca^2+^]_i_ changes are very fast and are almost entirely because of Ca^2+^ entry through NMDA receptors at the time when the Mg^2+^ block is relieved by the fast AMPA-mediated EPSP; (4) the [Ca^2+^]_i_ changes evoked by uncaging glutamate are slower than the changes evoked by synaptic release, suggesting that the relative contribution of Ca^2+^ entering through NMDA receptors at rest following uncaging is higher than following electrical stimulation.

## Significance Statement

Dendritic spines are the main sites where synaptically activated EPSPs are initiated and calcium changes are generated. Knowledge of these signals is critical for understanding synaptic integration and plasticity. We measured these changes in spines using high-speed fluorescence imaging with nonbuffering indicators following electrical stimulation. We found that sodium changes were primarily through AMPA receptors in spines and decayed by diffusion into dendrites with heterogenous times reflecting heterogeneous spine neck resistances. Calcium changes were fast and were primarily because of calcium entry through NMDA receptors opened when the magnesium block was relieved by the AMPA receptor mediated EPSP. We found differences between calcium signals evoked synaptically and signals evoked by two-photon glutamate uncaging, suggesting caution in interpreting these transients.

## Introduction

Dendritic spines have been at the center of many studies of synaptic transmission, especially those involving pyramidal neurons in the cortex and hippocampus. The magnitude and time course of synaptically activate voltage changes in spines are critical for determining how these synapses contribute to synaptic integration. Associated with these changes are changes in [Na^+^]_i_, which reflect Na^+^ entry through many channels, and are the major drivers of potential changes in spines. Similarly, the magnitude and time course of spine [Ca^2+^]_i_ changes are major determinates of synaptic plasticity and downstream signaling. Previous studies have examined these changes in dendritic spines in some detail (Yuste et al., 1999; Kovalchuk et al., 2000; Rose and Konnerth, 2001; Noguchi et al., 2005; Nevian and Sakmann, 2006; Bloodgood and Sabatini, 2007; Grunditz et al., 2008; Bloodgood et al., 2009; Holbro et al., 2010). However, there are some issues related to the approaches that were used, especially as related to the kinetics of the [Ca^2+^]_i_ and [Na^+^]_i_ changes, and which, in turn, may have implications for understanding synaptic integration and plasticity. One issue is whether two-photon glutamate uncaging, a technique used in many recent experiments (Noguchi et al., 2005; Araya et al., 2007; Bloodgood and Sabatini, 2007; Bloodgood et al., 2009), accurately mimics synaptic release of glutamate. Electrical stimulation releases glutamate from vesicles into a volume much smaller than an uncaging pulse does (Raghavachari and Lisman, 2004). This probably means that more widely distributed glutamate receptors with different sensitivities are activated by an uncaging pulse and for longer times than normal synaptic mechanisms. More physiological electrical stimulation has been used less frequently recently because it is hard to stimulate a single targeted spine (Allen and Stevens, 1994; Enoki et al., 2009). A second issue is whether recordings using relatively high affinity calcium indicators, like OGB-1 and members of the GCamp6 family (Chen et al., 2013), are accurate, even when allowing for the known nonlinearities and buffering by these compounds. A true measure of the kinetics and amplitude of the [Ca^2+^]_i_ change is important for determining the signaling steps downstream from the [Ca^2+^]_i_ increase. Measurements of physiological [Ca^2+^]_i_ changes using low-affinity indicators, with less buffering and less nonlinearity, have been difficult to achieve with adequate S/N using typical two-photon microscopes. A third issue is the relative importance of different membrane channels in the spine in generating the EPSP. The usual approach of measuring [Ca^2+^]_i_ changes or somatic voltages (Bloodgood and Sabatini, 2007) only indirectly relates to currents in the spine. Assays of the contributions of Na^+^ conductances and currents are missing.

To try to overcome these problems, we did experiments with minimal electrical stimulation to generate normal neurotransmitter release and detected [Ca^2+^]_i_ and [Na^+^]_i_ changes simultaneously with minimally buffering indicators using newly available focused single photon laser fluorescence stimulation without scanning and with a high-speed, sensitive CCD camera. With this approach, we found the following. (1) In mice, synaptic sodium current was almost entirely through AMPA receptors, with little contribution from entry through voltage-gated sodium channels (VGSCs) or NMDA receptor channels. A spectrum of sodium transient decay times was observed, suggesting a spectrum of spine neck resistances, even on the same dendrite. (2) Synaptic calcium signals had fast rise times, fast decay times, and large amplitudes, consistent with the major source of synaptic [Ca^2+^]_i_ increase being relief of the Mg^2+^ block of the NMDA receptors by the brief AMPA receptor mediated spine EPSP. The contribution of voltage-gated calcium channels (VGCCs) and Ca^2+^ entry through NMDA receptor channels at rest was clear but relatively small. (3) We compared the [Ca^2+^]_i_ changes evoked by synaptic stimulation with the transients evoked by two-photon glutamate uncaging using nonbuffering Ca^2+^ indicators. The changes evoked by uncaging had fast rise times, similar to the electrically evoked synaptic signals, but slower decays, suggesting that the contribution of Ca^2+^ entering through NMDA receptors at rest was higher following glutamate uncaging.

## Materials and Methods

### Slice preparation and electrophysiological procedures

Hippocampal slices (300-μm thick) from four- to 14-week-old C57BL/6 mice of either sex or four- to six-week-old Sprague Dawley rats of either sex (only a few experiments) were prepared using protocols standard for our laboratory (Miyazaki and Ross, 2015; Miyazaki et al., 2019). All procedures were approved by the institutional IACUC committee at New York Medical College. Submerged slices were placed in a chamber mounted on a stage rigidly bolted to an air table and were viewed with water-immersion lenses in an Olympus BX50WI microscope mounted on an X–Y translation stage. For maximum light detection and spatial resolution, we used an Olympus 60×, 1.1 NA lens. Even with this high NA lens, it was possible to patch neurons under visual control. Slices were superfused at 1 ml/min with standard artificial CSF (ACSF) consisting of the following (in mm): 124 NaCl, 2.5 KCl, 2 CaCl_2_, 1.0 MgCl_2_, 1.25 NaH_2_PO_4_, 26 NaHCO_3_, 0.01 d-serine, and 10.1 glucose. Somatic whole-cell recordings were made using patch pipettes pulled from 1.5-mm outer diameter thick-walled glass tubing (1511 M, Friedrich & Dimmock). Tight seals on CA1 pyramidal cell somata were made with the “blow and seal” technique using video-enhanced DIC optics to visualize the cells (Stuart et al., 1993). At the end of the experiment this video camera (Lumenera, INFINITY3S-1URM) was also used to take high resolution fluorescence images of the cell, using wide field LED excitation. For most experiments the pipette solution contained the following (in mm): 134 potassium gluconate, 4 NaCl, 6 KCl, 4 Mg-ATP, 0.3 Na-GTP salt, and 10 HEPES, pH adjusted to 7.2 with KOH. This solution was supplemented with different indicator combinations, which were matched with pairs of lasers. In most experiments we used sodium indicators SBFI (2 mm) or ING-2 (400 μm) combined with calcium indicators OGB-5N (150 μm), OGB-1 (50 μm), or fluo-5F (300 μm). The switch from the previously used bis-fura-2 (Miyazaki and Ross, 2017) to the low-affinity indicator OGB-5N was designed to reduce calcium buffering and increase linearity. Experiments were done at ∼30°C. All indicators were obtained from Invitrogen (now Thermo Fisher) except ING-2 (previously ANG-2), which was obtained from IonIndicators, LLC. Other drugs were obtained from Sigma-Aldrich and Tocris Bioscience.

### Data taking and analysis

For simultaneous sodium and calcium imaging we used the apparatus previously described (Miyazaki and Ross, 2015; Miyazaki et al., 2019). Briefly, fluorescence signals corresponding to changes in [Ca^2+^]_i_ and [Na^+^]_i_ were generated by exciting the corresponding indicators by rapidly switching focused laser beams every millisecond (SBFI with the 377-nm laser, OGB-1 and OGB-5N with the 476-nm laser, and ING-2 with the 517-nm laser; Vortran, Versalase, 60- to 80-mW peak power) and were detected by a high-speed (1000/2000 Hz), sensitive CCD camera (RedShirtImaging NeuroCCD-SMQ). The laser pulses were 200 μs wide and were timed to be in the center of the 1-ms camera frames. There was no bleed through from one frame to the next since tests showed that stopping individual lasers eliminated all the signals in the corresponding camera frames. Laser flashes were focused onto a single core optical fiber. The output light from the fiber was brought into a side port of the microscope and focused onto the slice. When we used a 200-μm diameter optical fiber and the 60× lens the laser spot on the slice was 13-μm diameter. Even with the rapid switching protocol and the high camera frame rate, the RMS noise of the steady state signal for a small region of interest (ROI) was proportional to the square root of the mean intensity (data not shown), indicating that the S/N of the measurements was shot noise limited. Although the camera lacks the *z*-axis resolution of a two-photon based detector, there was not much contribution from out of focus elements since only a few dendrites in the image field were filled with indicator. This system was enhanced using an ARM-based microcontroller (STM32 Nucleo-L476RG) to generate the pulse program driving the different lasers. The better S/N of these new measurements, compared with previous experiments (Miyazaki and Ross, 2017), was probably because of the use of higher intensity laser fluorescence excitation and narrower excitation area with less background fluorescence compared with the lower intensity and more widespread excitation with LEDs. An additional 1× to 2× magnifier was placed in front of the camera. Depending on the magnifier setting, the field of view was 32 × 32 or 16 × 16 μm^2^ when using the 60× objective. With these magnifications each of the 80 × 80 pixels of the camera covered 0.4 × 0.4 or 0.2 × 0.2 μm^2^. Experiments were under the combined control of Turbo-SM software, which came with the RedShirt camera, and custom MATLAB routines. These programs determined the frame rate and resolution of the camera (typically 80 × 80 pixels at 1000 Hz, i.e., 500 Hz in the Na^+^ and Ca^2+^ channels), synchronized the recording of electrical and optical signals, controlled the initiation of the laser pulse sequence, and triggered a Master-8 pulser, which in turn controlled the timing and duration of intrasomatic pulses and activated a synaptic stimulation protocol in most experiments. Optical and electrical data were recorded with 16 bit accuracy. Electrical data were recorded at 32,000 Hz. The resulting optical and electrical data were then processed through a custom MATLAB program, SCANDATA, written in our laboratory (available from the authors). This program separated the two data streams and aligned the sodium and calcium signals with voltage and current recordings from the soma. The optical signals were corrected for nonconstancy of the laser intensity during a sweep by normalizing to the intensity at the corner of the image field, away from the imaged dendrite. For some traces, an additional, smaller correction for indicator bleaching was applied by normalizing to the intensity during a sweep without stimulation or by subtracting a normalized trace fitted to a part of the recording without a signal. For the deconvolution calculation shown in Figure 5, we followed the procedure of Bloodgood et al. (2009). With careful attention to avoid unnecessary illumination of the slice, we typically were able to make over 10 trials of 200–300 ms in duration before noticing signs of photodynamic damage. Further evidence for the lack of significant photodynamic damage is the consistency of the results over many experiments and with different stimulation conditions (see Fig. 4*B–D*).

### Generation of synaptic responses

In most experiments synaptic responses were evoked with a theta-glass electrode filled with ACSF. An indicator-filled dendrite was positioned in the center of the RedShirt camera field and the stimulating electrode was advanced to ∼20 μm from the dendrite. The image of SBFI fluorescence was used for this procedure since it is brighter than OGB-5N at resting potential. From this position near the dendrite, weak electrical stimulation (200 μs in duration) often evoked a localized calcium response identified as a spine response if its size was <1 μm. If a response could not be evoked, the field and stimulating electrode were moved to a different dendrite, and the process was repeated. After detection of an optical response, we lowered the stimulation intensity until the minimal optical signal was detected.

### Glutamate uncaging

For uncaging experiments, the setup was integrated with an ultra-fast pulsed titanium-sapphire laser tuned to 720 or 725 nm (Chameleon Ultra, Coherent Inc.). The microscope was fixed in position and the stage with mounted micromanipulators was controlled with differential micrometers (Newport). The expanded beam of the laser was reflected off a dichroic mirror (FF700/SP, Semrock) and overfilled the back aperture of the 60× objective. The light intensity and duration of the laser pulse (typically 0.5–1.0 ms) were controlled by a Pockels Cell (Model 350–80, Conoptics Inc.) gated by a mechanical shutter with a 4-ms window. The shutter blocked the small amount of light (<15%) that came through the Pockels cell in the “off” setting. The stationary laser spot [∼0.7 μm, full-width at half-maximum (FWHM)] was positioned in the center of the field of view. Using the fluorescence of coinjected Alexa Fluor-594 (50 μm), we positioned a dendritic spine just next to this location. We estimate that the center of the uncaging spot was ∼1 μm from the edge of the spine. Caged MNI-glutamate (∼2.5–5.0 mm; R&D Systems) added to normal ACSF was bath applied through a recirculating system that had a volume of ∼6 ml. The flash intensity was adjusted to generate an EPSP of ∼1 mV. The amplitudes of the calcium responses were not critical for these experiments since we mostly were interested in the time course of the calcium transients. Transients were measured using either 150 or 300 μm OGB-5N. No difference was observed or expected since backpropagating action potential (bAP)-evoked transients measured with 600 μm OGB-5N had the same fast recovery times as those measured using 150 μm (see Results), indicating that there was no significant buffering at these concentrations.

Typically, recordings were made from one to four pyramidal neurons per animal. Cells were accepted for analysis only if dendrites were close to the surface of the slice and resting potential was below −55 mV. All example traces are from single trials (with one exception) with no temporal filtering. Pseudocolor images of the locations of putative spine signals were spatially smoothed using Matlab subroutine *imfilter*. The number of trials for each experiment was limited by photodynamic damage. Errors in the measurements are presented as SD. *p*-values of comparisons were calculated using paired *t* test in Figures 3 and 6 and unpaired *t* test in Figure 7.

## Results

### Identification of single spine sodium and calcium signals

**Figure 1.**
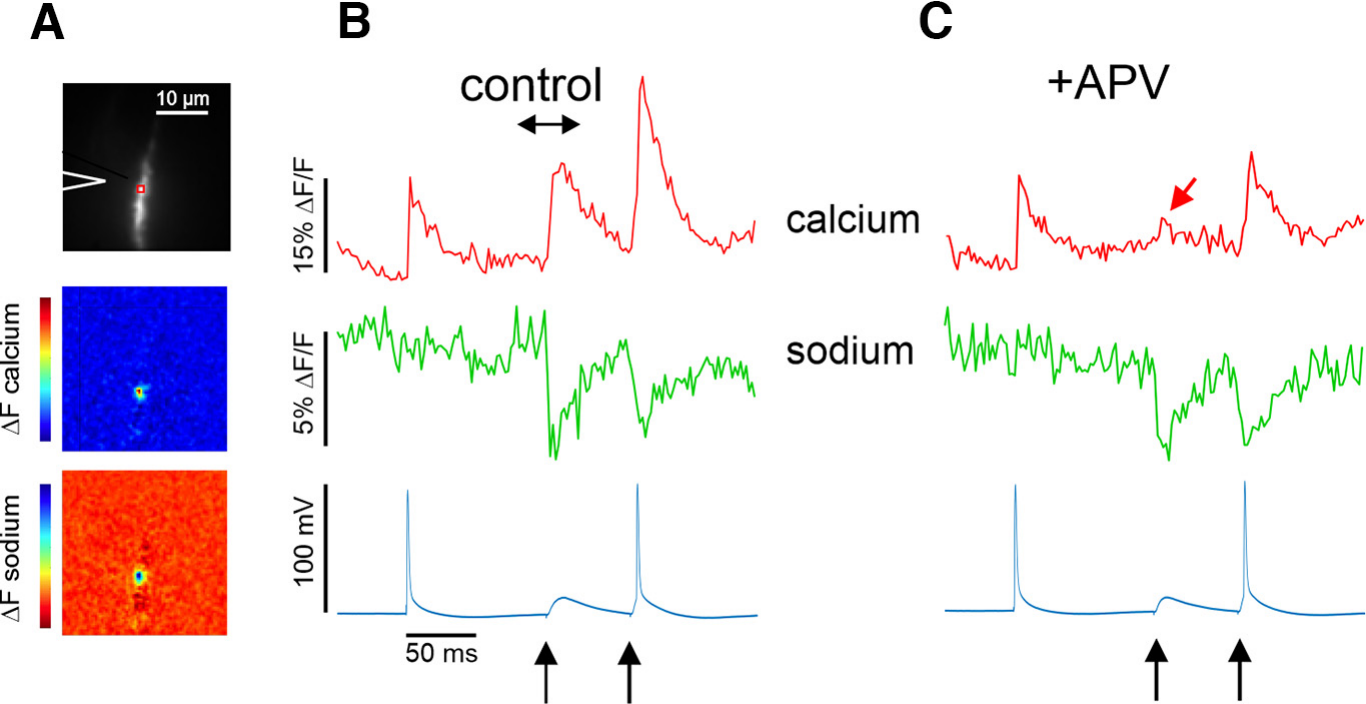
[Ca^2+^]_i_ and [Na^+^]_i_ changes in a dendritic spine evoked by a bAP and by synaptic stimulation. ***A***, Top, Fluorescence image of a dendritic segment with an ROI and the position of the stimulating electrode marked. Middle, Difference image (between the times at the ends of the double arrow in ***B***) of the [Ca^2+^]_i_ change (ΔF) evoked by synaptic stimulation (first arrow at the bottom of ***B***). The ROI was positioned over the location of the [Ca^2+^]_i_ change. Bottom, Difference image of the [Na^+^]_i_ change. The location of the change is the same as the location of the [Ca^2+^]_i_ change. The length of both the calcium and sodium spots was 0.8 μm (FWHM). The color bars on the side show the range of values in the image from maximum to minimum. These color bars also apply to Figures 2 and 7. ***B***, [Ca^2+^]_i_ and [Na^+^]_i_ changes at the ROI in response to an intrasomatically evoked bAP and two synaptic stimuli; the second synaptic response was suprathreshold. [Na^+^]_i_ increases are shown downward because SBFI fluorescence decreases with increasing [Na^+^]_i_. ***C***, Responses to the same stimuli with 100 μm APV added to the ACSF. The [Na^+^]_i_ changes were almost unaffected but most of the [Ca^2+^]_i_ increase was blocked, leaving only a small signal (red arrow).

In a typical experiment, we simultaneously measured the spine sodium and calcium transients in response to electrical stimulation. For the experiment shown in Figure 1, we first elicited a bAP in a pyramidal neuron with a 1-ms pulse in the soma. This pulse was followed by two synaptic stimuli separated by 50 ms. Although the stimulation pulses were equal, the second response reached threshold for generating an action potential. This protocol was then repeated in the presence of 100 μm APV, which blocked NMDA receptors. This kind of experiment was repeated many times in different cells. The responses in this cell were particularly clean, providing a strong template for further analysis.

We first made a difference image of the fluorescence changes corresponding to the [Ca^2+^]_i_ and [Na^+^]_i_ changes elicited by synaptic stimulation (start and stop times indicated by double headed arrow over the synaptic optical response in Fig. 1*B*). These revealed locations of [Ca^2+^]_i_ and [Na^+^]_i_ changes on the dendrite, as shown in the two lower images in Figure 1*A*. For both sodium and calcium signals these locations were small spots about the size of a dendritic spine (average size ∼0.7 ± 0.2 μm, FWHM, measured with no spatial filter; *n* = 18). Using the camera as a detector allowed us to search for the activated spines after the trial was recorded and could determine, in most cases, if more than a single spine was activated in the illuminated region on the dendrite. In the example shown in Figure 1, only one spine in the image field (32 × 32 μm^2^) was activated, although from the amplitude of the EPSP in the soma (∼5 mV) we know that multiple spines in the dendrites must have been activated (Magee and Cook, 2000). In this dendrite, we could not see the activated spine, possibly because it was projecting above or below the dendrite. But its size, and the isolated response, strongly suggests that only one spine on this dendritic segment was activated. We then placed a small analysis box (ROI) over the spot and measured the dynamic [Ca^2+^]_i_ and [Na^+^]_i_ changes from that location. In this way we analyzed calcium signals from 66 isolated spines [OGB-5N (51), Fluo-5F (15), most from mice (35; OGB-5N (20), Fluo-5F (15)]; the remainder were from rats. Most of these responses also had localized sodium signals. Ten responses [OGB-5N (9), Fluo-5F(1)] were further identified as signals from single spines because we could clearly see the spine where the signal came from. This was probably because the spine projected to the side of the dendrite and the dendrite was close to the surface of the slice, making for a sharper image with the camera.

### bAP-evoked signals

To establish a reference for synaptic responses we first measured the [Ca^2+^]_i_ and [Na^+^]_i_ changes from bAPs using low-affinity indicators. In the experiment shown in Figure 1, the bAP elicited a rapid calcium transient, which rose to peak <2 ms, the effective frame interval for most calcium measurements. In eight cells, the average rise time was also <2 ms, as in previous experiments with the same time resolution (Sabatini et al., 2002). To examine the rise time of these signals more accurately we made a series of measurements at 4× higher speed (2000 Hz) without simultaneous sodium imaging. With this higher precision the 10–90% rise times were 1.58 ± 0.14 ms (*n* = 4) in the proximal dendrites, consistent with previous measurements (Jaafari et al., 2014). The same values were measured in the distal, and oblique dendrites. The half decay time of the bAP signal in the dendrites measured at this speed was 20.5 ± 5.2 ms (*n* = 4). When measured at 500 Hz the half recovery time in the spine was 12.0 ± 5.2 ms (*n* = 13) and 17.6 ± 3.6 ms (*n* = 9) in the nearby dendrites. These decay times, measured with 150 μm OGB-5N, are comparable to the half decay times measured previously (Sabatini et al., 2002) with very low concentrations of higher affinity indicators (OGB-1, fluo-4, Mg-Green). This concentration of OGB-5N was not buffering the calcium in the spine since similar half decay times in the dendrites were measured with 600 μm OGB-5N (18.6 ± 2.4 ms; *n* = 3). This fact, together with the observation that the magnitudes of all evoked calcium transients (see below) are far below the K_d_ of OGB-5N (∼40 μm), means that the fluorescence decay times measured in the experiments in this paper are an accurate reflection of the underlying calcium dynamics and are not sensitive to the level of indicator filling in the dendrites and spines.

**Figure 2.**
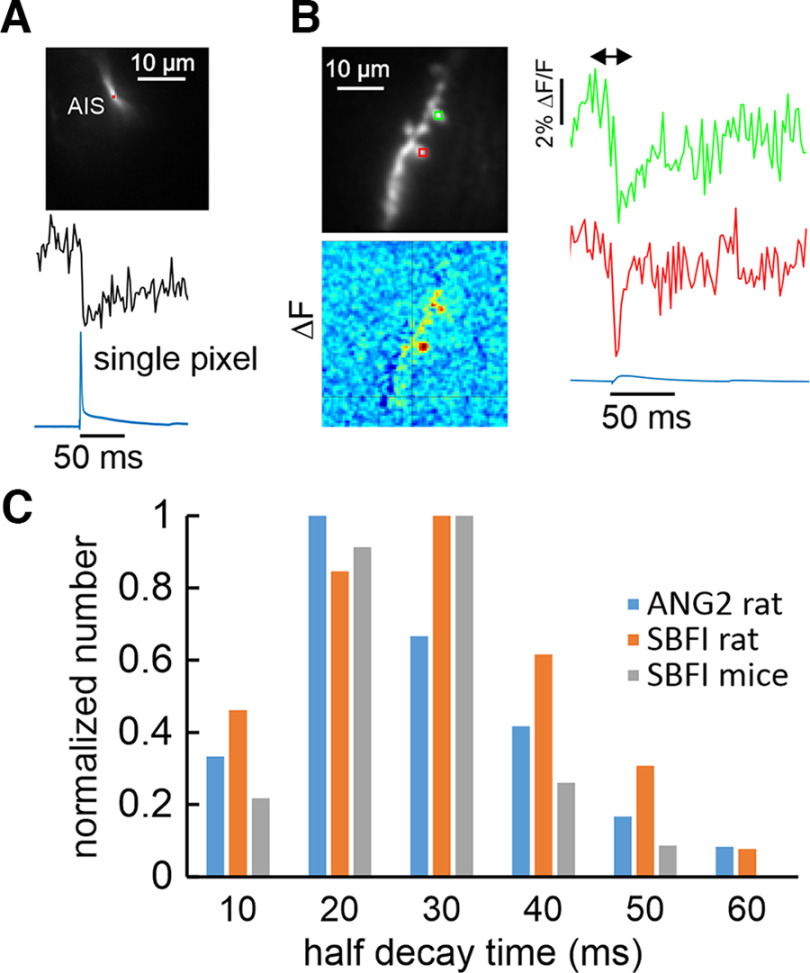
Sodium transients in dendritic spines and the axon initial segment. ***A***, SBFI fluorescence transient detected by a single pixel (red box) covering an area of 0.4 × 0.4 μm^2^ in the axon initial segment (AIS) in response to an intrasomatically evoked AP (blue trace). The rise time of the signals was <2 ms (the frame interval of the camera) and was easily detected without averaging or filtering. ***B***, An example of two spine signal with fast (4.1 ± 1.2 ms) and moderate (31.7 ± 17.2 ms) Na^+^ half recovery times activated by the same extracellular stimulus (calculated from exponential fits to the transients). Fluorescence signals from the two ROIs on a mouse pyramidal neuron and the corresponding somatic electrical recording are shown. ***C***, Distribution of half decay times of spine Na^+^ transients in response to single synaptic stimuli. Measurements were made from 20 mouse spines (57 traces) using SBFI, 20 rat spines (43 traces) using SBFI, and 14 rat spines (36 traces) using ING-2. Half decay times were binned in 10-ms groups and normalized to the peak number for each protocol. The distributions were similar among the three groupings. Recordings with ING-2 from Miyazaki and Ross (2017).

A sodium response in the spine to a single bAP was not detected using SBFI (*n* > 20). Since the [Ca^2+^]_i_ change from the bAP in the spine in Figure 1 (and all other spines selected for analysis) was clear we know the spike reached the spine. In three additional cells we averaged the response to trains of 40 bAPs and looked for spine sodium signals; none were detected. Figure 2*A* shows that, with the same apparatus, we easily detected an action potential-evoked [Na^+^]_i_ increase from an area of 0.4 × 0.4 μm^2^ (comparable to spine dimensions) in the axon initial segment, which is known to have a higher density of Na^+^ channels (Kole et al., 2008; Fleidervish et al., 2010) than dendrites (Stuart and Sakmann, 1994; Magee and Johnston, 1995). Previous experiments on rats showed that small [Na^+^]_i_ increases could be detected from large dendritic segments from trains of bAPs with signal averaging (Rose et al., 1999; using SBFI; Miyazaki and Ross, 2017; using ING-2), This result is not a difference between rats and mice since additional experiments using ING-2 (data not shown) detected small [Na^+^]_i_ changes in large segments of murine dendrites with averaging of multiple bAP signals.

### Na^+^ signals from synaptic stimulation

In contrast to the bAP result, the middle panel of Figure 1 shows that a spine [Na^+^]_i_ increase was clearly detected following single synaptic stimulation. In general, while [Na^+^]_i_ increases in spines from single bAPs were never detected, synaptic responses were often detected. This result was found from spines within 150 μm from the soma, where bAP amplitude is expected to be large (Spruston et al., 1995b), and from spines in the most distal dendrites, where the bAP amplitude is smaller and the EPSP is bigger (Magee and Cook, 2000).

The kinetics of the synaptically activated [Na^+^]_i_ increases on mouse spines was consistent with previous measurements on rat pyramidal neuron spines (Miyazaki and Ross, 2017). For these new mouse experiments, we measured the spine [Na^+^]_i_ changes with SBFI at higher time resolution (500 Hz) and improved S/N. The 10–90% rise time of the [Na^+^]_i_ increase was 7.0 ± 4.5 ms (*n* = 13). This rise time is slower than the <2-ms rise time of the AP-evoked signal in the axon initial segment (*n* = 6; data not shown; see also Filipis and Canepari, 2021), showing that the slower rise time is not because of technical factors like camera response time or indicator rate constants. It likely reflects the integral of the sodium current underlying the fast EPSP in the spine (Palmer and Stuart, 2009; Popovic et al., 2015), which is slower than the duration of the current. The [Na^+^]_i_ change in response to the second synaptic stimulus was almost identical, although the response was suprathreshold, evoking a bAP. As with the response to the bAP alone, there was no obvious contribution of the synaptically evoked bAP to the [Na^+^]_i_ increase, in contrast to previously reported results (Rose and Konnerth, 2001). The larger spine [Ca^2+^]_i_ increase, when the spike was evoked, confirmed that the bAP reached this location. These results indicate that activation of voltage-gated sodium channels (VGSCs) make little contribution to the synaptically evoked [Na^+^]_i_ increase in spines (see also Miyazaki and Ross, 2017). The kinetics of the sodium signal were almost unaffected by 100 μm APV (rise time reduced from 6.6 ± 3.6 to 6.0 ± 2.7 ms; *p* = 0.5; *n* = 4; half decay time reduced from 22.8 ± 4.5 to 19.7 ± 1.8 ms; *p* = 0.4; *n* = 4). The amplitude (%ΔF/F), while hard to measure because of the background fluorescence, was reduced from 8.8 ± 3.0 to 7.6 ± 2.4 (*p* = 0.4; *n* = 4). Since almost all the [Na^+^]_i_ transient remains in APV, most of the influx underlying the signal does not come though NMDA receptors, although there must be some contribution through this pathway since >93% of the current through NMDA receptors is carried by sodium (Schneggenburger et al., 1993). Most likely the influx is through AMPA receptors, since most of it is blocked by NBQX (Fig. 5). This conclusion is consistent with the observation that the EPSP, as recorded in the soma, was almost unaffected by APV (Fig. 1), an observation reported by many groups (Feldmeyer et al., 2002).

**Figure 3.**
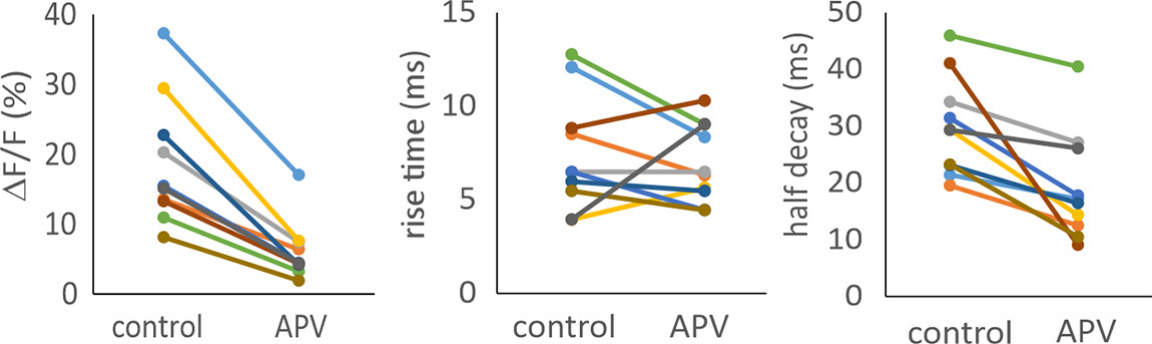
The effect of APV (100 μm) on synaptically evoked Ca^2+^ transients in dendritic spines. APV reduced the magnitude to 0.33 ± 0.09% of control (*p* = 0.00,006) and half decay times from 30.2 ± 8.6 to 18.7 ± 9.2 ms (*p* = 0.003) but had no significant effect on the rise times (91% of control; *p* = 0.5). Changes for each of ten spines are shown.

**Figure 4.**
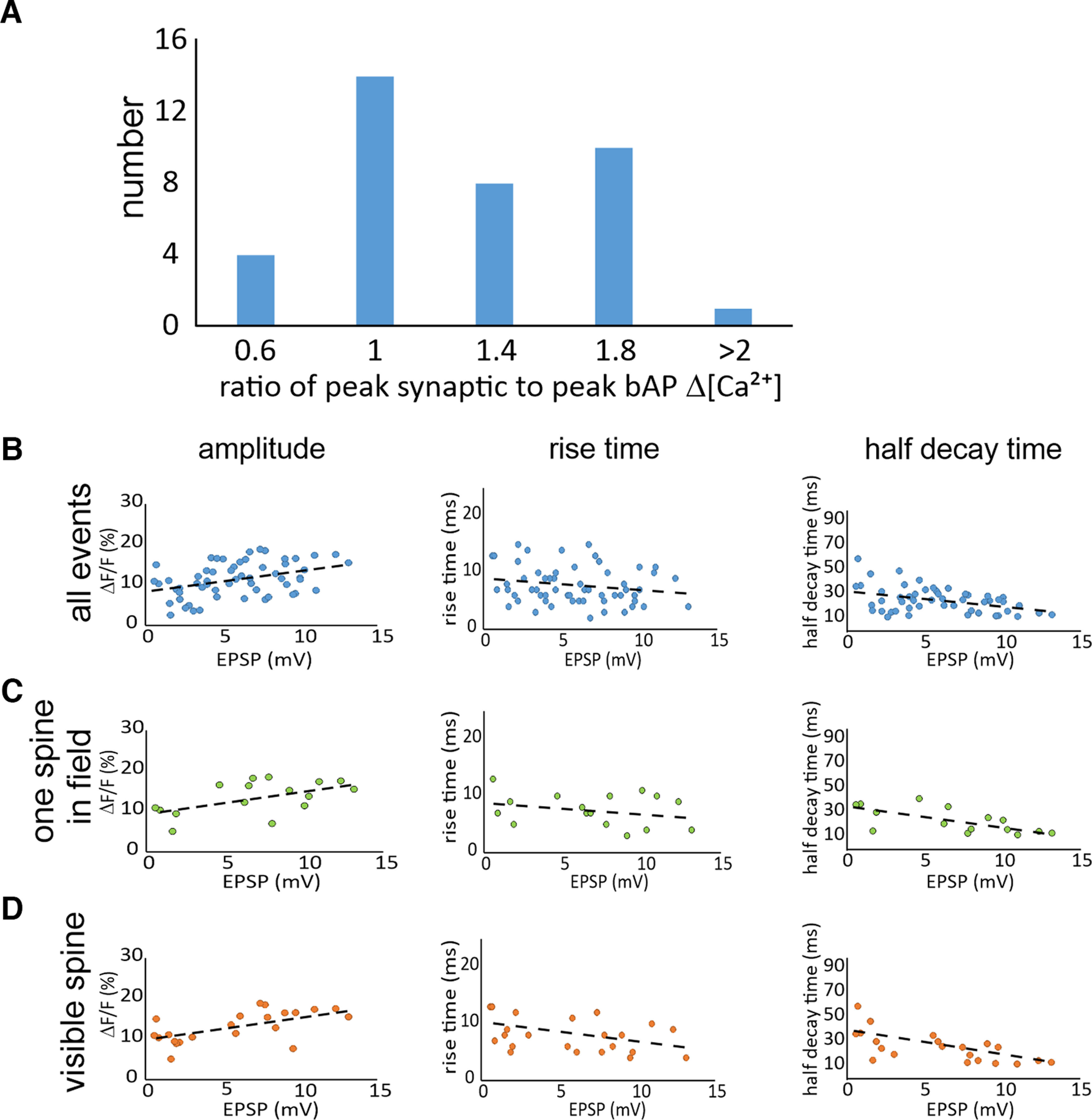
Magnitudes and time courses of synaptically evoked Ca^2+^ transients. ***A***, Histogram of the number of traces with different values for the ratio of the synaptic [Ca^2+^]_i_ increase to the [Ca^2+^]_i_ increase evoked by a bAP at the same spine location. Events were binned in five groups (*n* = 40 trials from 24 spines). ***B***, Distributions of the ΔF/F magnitudes, 10–90% rise times, and half decay times of synaptically evoked Ca^2+^ transients in spines as a function of the somatically recorded EPSP amplitude. All parameters were measured using 150 μm OGB-5N. No correction was made for background fluorescence (*n* = 58 total trials from 20 spines). ***C***, Distribution of signals where only one active spine was visible in the camera field (16 trials from single activated spines in the field of view from 10 spines). ***D***, Distribution of signals from the subset of responses from clearly visible spines (16 trials from 8 spines from 8 cells). The dashed lines in each panel show the best linear fits to the data. In most of these cells, the amplitude of the EPSP suggests that multiple axons were stimulated. The responses in one cell where only a single presynaptic fiber was activated is shown in Extended Data Figure 4-1.

A spectrum of synaptically activated [Na^+^]_i_ half decay times was measured in murine pyramidal neurons with 2 mm SBFI. An example of transients in two spines on the same dendrite with fast and moderate Na^+^ recovery times, activated by the same stimulus, is shown in Figure 2*B*. The half recovery times ranged from 10–60 ms (mean 21.7 ± 9.6 ms; *n* = 57; Fig. 2*C*), with approximately the same distribution measured in rat pyramidal neurons with SBFI (mean 23.9 ± 12.2; *n* = 43) and with ING-2 (mean 20.0 ± 15.7; *n* = 33; Miyazaki and Ross, 2017). Two cells (data not shown) had very long Na^+^ transients, whose decay times could not be measured. The similarity of decay times measured with the two indicators is consistent with their being weak buffers with high K_d_ values for the sodium:indicator reaction (Miyazaki and Ross, 2017). If the indicators did buffer sodium, the recovery times of the true sodium transients would be even faster. Similar decay time distributions were measured from both rat and mouse spines (Fig. 2*C*), suggesting that these are general properties, at least among rodent spines. The rapid decay times are because of Na^+^ diffusion from the spine to the dendrite as shown previously for spines in rat pyramidal neurons (Miyazaki and Ross, 2017). Similar fast removal times were measured from other localized sites of Na^+^ entry in the axon initial segment and node of Ranvier (Fleidervish et al., 2010). In those axon experiments, the fast recovery times were not affected by ouabain, a blocker of Na/K-ATPase, showing that for short times the removal was not because of a pump. In other conditions, when there is no significant [Na^+^]_i_ gradient, e.g., in the dendrites following a train of bAPs, Na^+^ is removed slowly (seconds) via a temperature-sensitive membrane pump (Rose et al., 1999). In this situation, removal by diffusion is less significant, since there is expected to be little [Na^+^]_i_ gradient in the dendrites following bAPs. The fast removal time of Na^+^ by diffusion from spines is coincidently similar to the removal time of synaptically evoked Ca^2+^ (Figs. 3, 4*B*) but through an entirely different mechanism. Ca^2+^ is removed through a combination of pumps and buffers (Scheuss et al., 2006) and not by diffusion to the dendrites (Sabatini et al., 2002).

10.1523/ENEURO.0396-22.2022.f4-1 Extended Data Figure 4-1 An example of a spine where synaptic calcium signals were probably evoked by activating a single presynaptic axon. The top image shows a low magnification picture of the pyramidal neuron filled with fluorescent indicator. The position to the stimulating electrode is shown. The dotted box indicates the region examined at higher magnification in the image below. The ROI is indicated with a small red box. The difference image of the evoked Ca^2+^ signal (as in Fig. 1) is shown below. The traces on the right show 14 calcium signals and somatic electrical recordings in response to single weak electrical shocks. Five traces, marked with an asterisk, have both electrical and optical responses; the others did not respond (see also Enoki et al., 2009) Using this assay, we concluded that only a single presynaptic fiber was activated, with stochastic success in releasing transmitter. For these five active responses the average EPSP amplitude was 0.85 ± 0.24 mV; the average calcium rise time was 11.4 ± 2.5 ms, and the average half decay time was 31.4 ± 10.9 ms. Download Figure 4-1, file.

The variations in Na^+^ decay times were greater than the error in measuring the times. In two spines where we were able to measure the signal many times, we found that the half decay times were 22.2 ± 5.4 (*n* = 7) and 28.1 ± 7.3 (*n* = 7) ms; the errors are much less than the range of half decay times. Since the somatic EPSP and the spine [Na^+^]_i_ increase were almost unaffected by APV the spine EPSP also should be unchanged in APV. Therefore, these different decay times reflect real physiological variation. Most likely they are because of variations in spine properties, like neck length, but some part of the slower signals may be because of a contribution from a dendrite included in the same ROI where diffusion from the spine slows the response. Without this component (unknown) the recovery times would be even faster.

### Ca^2+^ signals from synaptic stimulation

The calcium response to synaptic stimulation was superficially similar to the bAP signal (Fig. 1*A*). However, careful examination showed that the 10–90% rise time was slower (7.9 ± 3.3 ms; *n* = 20 spines) and the half decay time (25.4 ± 11.6 ms; range 15–50 ms; *n* = 18 spines) was slower than the bAP signal. As was shown many times previously (Yuste et al., 1999), most of the [Ca^2+^]_i_ increase was because of entry through NMDA receptor channels, since the addition of 100 μm APV reduced the Ca^2+^ signal to undetectable levels in many cells when OGB-5N was used (*n* = 8). However, it is possible that some of these nonresponses were because of failure of transmitter release. In nine cells where a clear signal in APV was detected (Fig. 3), the remaining signal amplitude was ∼32% of the control signal and is likely to reflect Ca^2+^ entry through VGCCs (Bloodgood et al., 2009). One example is shown in Figure 1, arrow. The half decay times of these remaining transients in APV (18.7 ± 9.2 ms) were faster than the synaptic response without APV (30.2 ± 8.6) and were comparable to the half decay times of the voltage-evoked bAP signals in spines (12.0 ± 5.2 ms). The fast rise times were unaffected by APV. This result suggests that both the NMDA receptor-mediated current and the remaining VGCC-mediated current are both driven by the same sharp voltage rise from the AMPA-mediated EPSP. This means that the small [Ca^2+^]_i_ increase in APV is about the magnitude of the VGCC signal in physiological conditions and is only a small fraction of the total synaptic [Ca^2+^]_i_ increase. When the synaptic response was suprathreshold for generating a bAP (Fig. 1) the [Ca^2+^]_i_ increase always was larger, reflecting the additional Ca^2+^ entry through VGCCs (*n* > 10). This was confirmed by the response to suprathreshold stimulation in APV. In this case, the time course of the Ca^2+^ transient was almost identical to the response to the bAP alone (Fig. 1*C*).

### Magnitude of synaptic [Ca^2+^]_i_ increase

The amplitude measurements cannot easily be calibrated in terms of the [Ca^2+^]_i_ change since there was significant background fluorescence. However, these amplitudes can be directly compared with the amplitudes of the bAP-evoked ΔF/F values in the same spine; the background levels are the same for both measurements. The distribution of these ratios is shown in the histogram in Fig. 4*A*; the average is 1.2 ± 0.5 (*n* = 40 trials from 24 cells). Significant variation is expected since spines are morphologically heterogeneous, the number of released vesicles varies from trial to trial, and the amplitude of the bAP varies in different dendritic regions. Previously, Sabatini et al. (2002), using two-photon microscopy, estimated the amplitude of bAP-evoked [Ca^2+^]_i_ increases, in the limit of zero added indicator, as 1.7 ± 0.6 μm. These measurements were not contaminated by background fluorescence because of the high *z*-axis resolution of the two-photon process. Using their value as a reference suggests that the average synaptically evoked [Ca^2+^]_i_ increase was 2.0 ± 0.8 μm (1.7 × 1.2). This is the expected value without indicator since 150 μm OGB-5N is not a significant buffer.

One potential correction to this calculation for some events is that the ROI for the spine might include some underlying or overlying dendrite. In this case, the dendritic fluorescence would be background for the synaptic signal but would be an active signal source for the bAP signal. To try to correct for this error we selected spines that were clearly visible to the side of the dendrite (Fig. 2*B*). For these events the dendrite should make little contribution to a signal with an ROI placed over the spine. For five cells that met this criterion we found a ratio of 1.1 ± 0.5, leading to an average peak [Ca^2+^]_i_ change of 1.9 ± 0.9 μm, about the same as determined from the full dataset.

### Possible effects of other spines

Since almost all the synaptically activated [Na^+^]_i_ increase is because of Na^+^ entry through AMPA receptors on the activated spine, which has little voltage dependence, and there is little voltage-gated Na^+^ entry, it is unlikely that activation of other spines by the stimulation current affects the localized Na^+^ signal in the spine, although some of the voltage from those other spines invades the measured spine. However, the contribution of VGCCs to the [Ca^2+^]_i_ increase in the spine, suggests that the voltage spread from other spines, could affect the magnitude and time course of the spine calcium signal (Palmer and Stuart, 2009). To investigate this possibility, we plotted the rise time, decay time, and amplitude of the [Ca^2+^]_i_ change, measured with 150 μm OGB-5N, as a function of the somatically recorded EPSP (Fig. 4*B*). We reasoned that although there is not a direct relationship between the somatic EPSP and the EPSP in the examined spine, there would be some correlation if spine EPSP amplitude was important in determining these signal parameters. There was a weak correlation of ΔF/F amplitude with EPSP amplitude. However, it is not clear whether the correlation is meaningful since the ΔF/F values were not corrected for background fluorescence and may be different for each spine. There was no correlation of rise time with EPSP size. There was a correlation with the half decay time of the synaptic Ca^2+^ transient, which became more rapid at higher EPSP amplitudes. This trend might reflect the greater contribution of VGCC-mediated Ca^2+^ entry at higher EPSP amplitudes. Increased Ca^2+^-activated K^+^ conductance might also affect the time course of the calcium signal.

It is possible that some of these conclusions depend on whether the spines were isolated in the camera field or had close neighbors that were activated at the same time. Also, the results might differ if the spines were visible at the side of the dendrite or were not clear because they were above or below the dendrite. To check on these possibilities we measured the results from trials with isolated spines and visible spines and plotted them separately. The patterns in each of the panels in Figure 4*C*,*D* show that there were no obvious differences correlated with how the events were selected.

The highest EPSP, ∼12 mV, probably reflects activation of at least 15 synaptic sites (Magee and Cook, 2000). The weak correlation of these parameters with the somatic EPSP amplitude suggests that the EPSPs generated in other spines had little influence on the EPSP in the analyzed spine, although we cannot be precise in this estimate. These other spines must have acted at some distance since there was either only one activated spine or widely separated spines in the camera field for most the data points in Figure 4*C*. Since the EPSP did spread to the soma, this result suggests that the spine EPSP amplitude is much higher than the somatic EPSP amplitude. In other related experiments, Carter et al. (2007) found only small interactions among nearby spines in striatal medium spiny neurons, and Bloodgood et al. (2009) found little interaction among pyramidal neuron spines. In contrast, Harnett et al. (2012) and Weber et al. (2016) reported significant interactions among spines.

A more direct way of eliminating the influence of other spines on the Ca^2+^ signal of the examined spine, is to stimulate only a single axon, which is hard to do. After some effort we had two successes; the best one is shown in Extended Data Figure 4-1. We found that the optical time constants evoked by this single axonal fiber (rise time: 11.4 ± 2.5 ms; half decay time: 31.4 ± 10.9 ms) were like those measured with activation of multiple spines (Fig. 4*B*) and the EPSP amplitude (0.85 ± 0.24 mV) was similar to those measured with single fiber activation in purely electrical experiments (Allen and Stevens, 1994).

### Analysis of the components of the calcium signals

**Figure 5.**
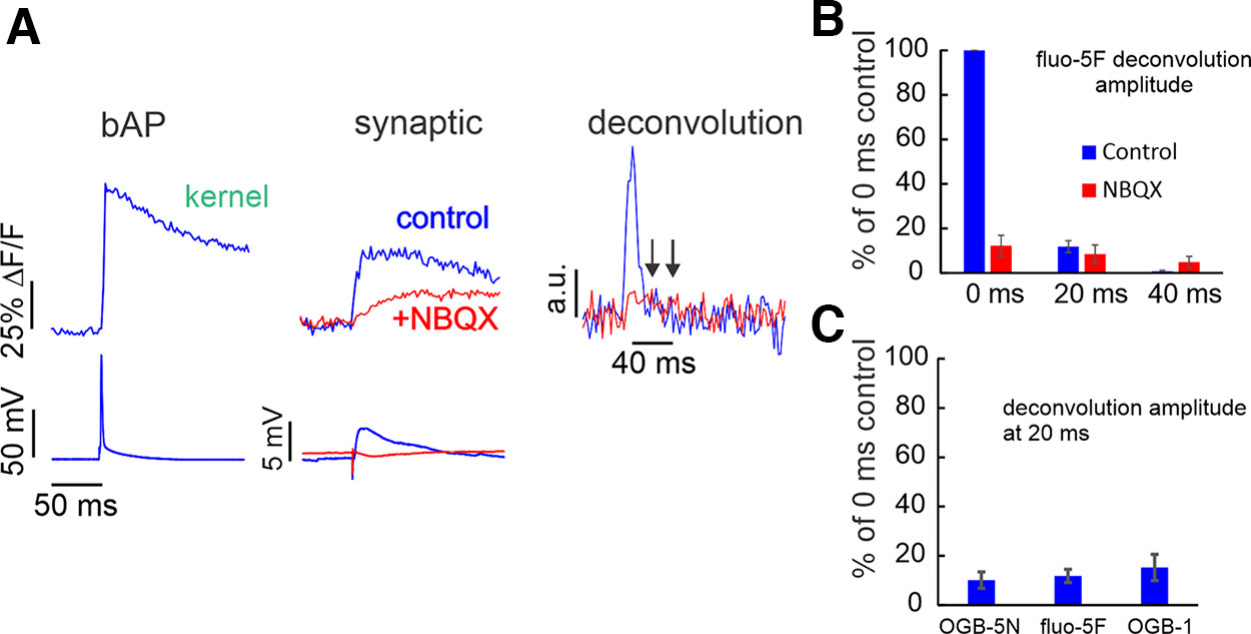
Synaptic Ca^2+^ currents analyzed by deconvolution of the Ca^2+^ transients using three indicators of different K_d_ values. ***A***, Left, bAP-evoked Ca^2+^ transient detected with fluo-5F used as the kernel in the deconvolution. Center, Responses with and without NBQX of synaptically evoked Ca^2+^ transients, using fluo-5F. In NBQX the response is smaller and slower. Right, Deconvolution of the synaptic responses using the bAP-evoked kernel. The largest response is in the first 10 ms after synaptic stimulation, and this component is blocked by NBQX. Arrows indicate 20 and 40 ms after the peak of the calcium current. ***B***, Normalized histogram of the Ca^2+^ currents in the two conditions binned in 20-ms groups. Most of the effect of NBQX is in the first bin (0–10 ms; *n* = 5). ***C***, Histogram of the effect of NBQX on the first 20 ms of the synaptic response, analyzed with the three indicators. The effects were similar (OGB-5N, *n* = 7; fluo-5F, *n* = 8; OGB-1, *n* = 2).

The rise times and decay times of the synaptically evoked calcium transients are faster than many previous measurements of these times. Nevian and Sakmann (2004) and Enoki et al. (2009) measured fast rise times although they used high-affinity indicators, which may have distorted the responses. In our experiments using OGB-5N, most rise times were <10 ms, which is much faster than the duration of the NMDA receptor conductance when there is no Mg^2+^ block (Spruston et al., 1995a) This conclusion suggests that a large part of the calcium signal is driven by the fast EPSP in the spine, which briefly relieves the Mg^2+^ block. This idea, which has been suggested previously (Bloodgood et al., 2009; Holbro et al., 2010), does not reveal which fraction of the [Ca^2+^]_i_ increase results from this mechanism, and which part results from Ca^2+^ entering through the NMDA receptor after the EPSP is finished and the membrane potential returns to resting level (Kovalchuk et al., 2000). One way to separate these two contributions is to deconvolve the synaptic calcium signal into temporal components, using the [Ca^2+^]_i_ transient from a bAP or other brief voltage-gated Ca^2+^ signal as a template. Bloodgood et al. (2009) used this approach to analyze the components of signals evoked by two-photon glutamate uncaging. They found that an early phase of Ca^2+^ entry was driven by the spine AMPA-mediated EPSP since it was blocked by the AMPA receptor antagonist NBQX. However, most of the signal was because of the second slow component resulting from Ca^2+^ entry through the NMDA receptor at resting potential. We used this method to analyze the synaptically evoked Ca^2+^ transients. We used the Ca^2+^ signal from the bAP as the impulse current (kernel) and deconvolved the synaptic signal in experiments using different calcium indicators [150 μm OGB-5N, 300 μm fluo-5F (to match conditions in previous published experiments), and 50 μm OGB1]. The bAP signal and the synaptic signal in normal ACSF and with 10 μm NBQX are shown in Figure 5*A*. NBQX blocks the fast component of the synaptic signal and almost all the somatically recorded EPSP. The deconvolution shows that most of the calcium influx occurred in the first 20 ms, and this component was almost eliminated in NBQX. Figure 5*B* shows that in experiments using fluo-5F the slow Ca^2+^ current (at 20 and 40 ms) comprised <10% of the early signal. Similar results were found with the other indicators (Fig. 5*C*). The results were qualitatively the same as in Bloodgood et al. (2009), although we found that the early synaptic component was significantly larger than the fast component recorded in their two-photon uncaging experiments. The difference was not because of different deconvolution techniques since we reproduced the deconvolution records of Bloodgood et al. (2009) when we used their recorded calcium transients in our deconvolution calculations.

**Figure 6.**
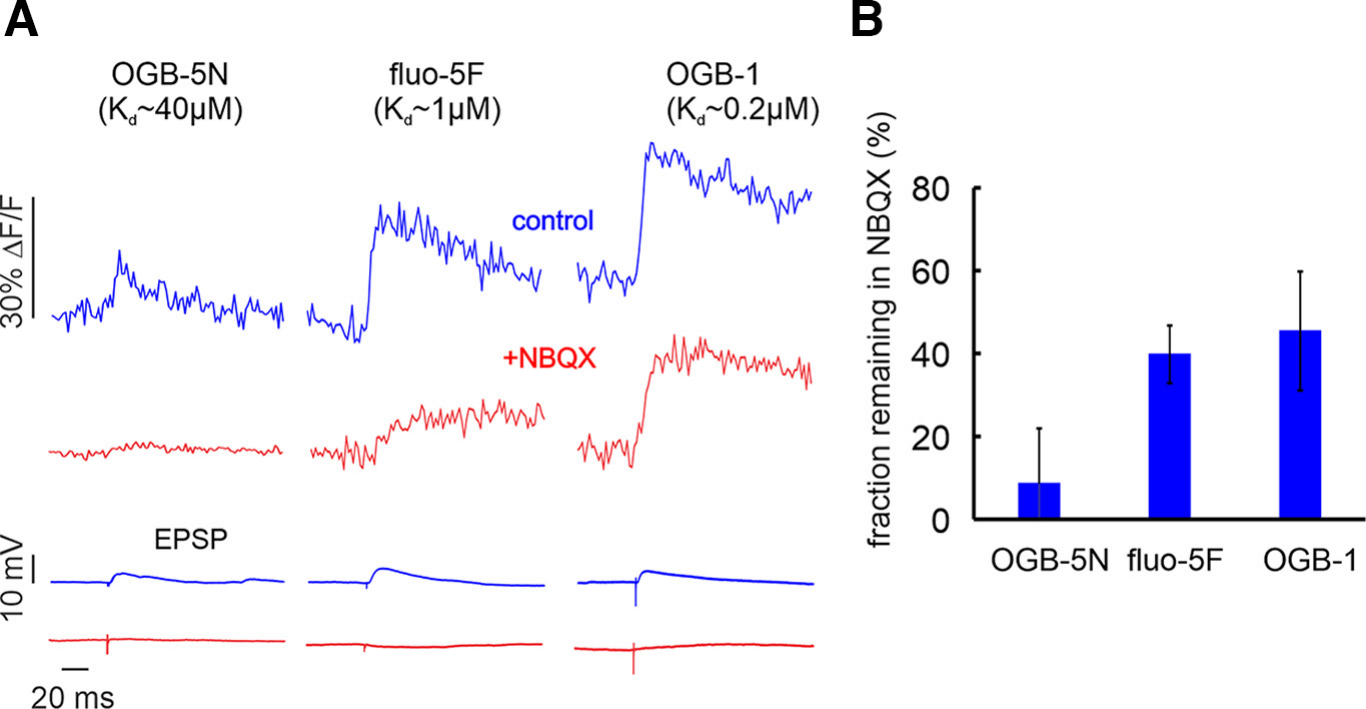
The effect of NBQX on synaptically evoked Ca^2+^ transients detected with indicators of different K_d_ values. ***A***, Examples of responses with and without NBQX. The effect of NBQX is greatest when OGB-5N was used. All records are single trials except the recording of NBQX on the OGB-5N cell (average of 3 traces). The effect of NBQX on the somatically recorded EPSP is shown below. ***B***, Histogram of the effect of NBQX on the amplitudes of the synaptic transients detected with the three indicators. The fractional reduction is greater with OGB-5N than with fluo-5F (*p* = 0.004) or with OGB-1 (*p* = 0.001; OGB-5N, 150 μm, *n* = 5; fluo-5F, 300 μm, *n* = 5; OGB-1, 50 μm, *n* = 5).

These experiments show that most of the synaptic signal recorded using fluo-5F, especially the fast component, was blocked by NBQX. Similar results were found in spiny stellate cells (Nevian and Sakmann, 2004). However, some experiments (Kovalchuk et al., 2000) suggest that similar AMPA receptor blockers like CNQX have little effect on the subthreshold synaptic calcium signal when 100 μm OGB-1 is used as the calcium indicator. One possible explanation for the difference is that synaptic responses using high affinity indicators like OGB-1, might saturate the indicator since the synaptic [Ca^2+^]_i_ increase was typically ∼2–3 μm (see above), while the K_d_ for OGB-1 is only 0.2 μm. Recordings with OGB-5N (K_d_ ∼40 μm) would not saturate (Higley and Sabatini, 2008). Another possible explanation is that Kovalchuk et al. (2000) used multiple stimuli to evoke a synaptic calcium response, which often evoke regenerative NMDA receptor responses. To examine these possibilities, we tested the effect of NBQX on the synaptic signals generated by single shocks using three different indicators with different K_d_ values. Figure 6*A* shows examples of these experiments. The recovery times of the synaptic calcium signals are fastest with OGB-5N, consistent with it being the least buffering indicator. More to the point, NBQX blocked a much higher fraction of the synaptic signal when OGB-5N was used. Summary statistics for this experiment are shown in Figure 6*B*. This result suggests that experiments using OGB-1, and to some extent fluo-5F, are missing the peak of the spine synaptic transient. When the less buffering indicator, OGB-5N, was used, it becomes clearer that most of the transient is blocked by NBQX.

### Comparison between synaptic and glutamate uncaging-evoked calcium transients

All the above experiments analyzing calcium transients used electrically evoked synaptic responses and OGB-5N to measure the calcium signals. But most recent experiments (Bloodgood et al., 2009; Holbro et al., 2010; Beaulieu-Laroche and Harnett, 2018) used glutamate uncaging to evoke the synaptic responses and relatively higher affinity indicators to measure the calcium transients. The responses we recorded were clearly faster than those recorded in most published uncaging experiments and the slow calcium current (20–40 ms) was less than slow current produced by two photon glutamate uncaging. But it is not clear how much the difference is because of the indicator and how much to the method of stimulation. To examine this issue, we did some basic glutamate uncaging experiments, following the protocols of other investigators, with the exception that we used OGB-5N to detect the Ca^2+^ transients.

**Figure 7.**
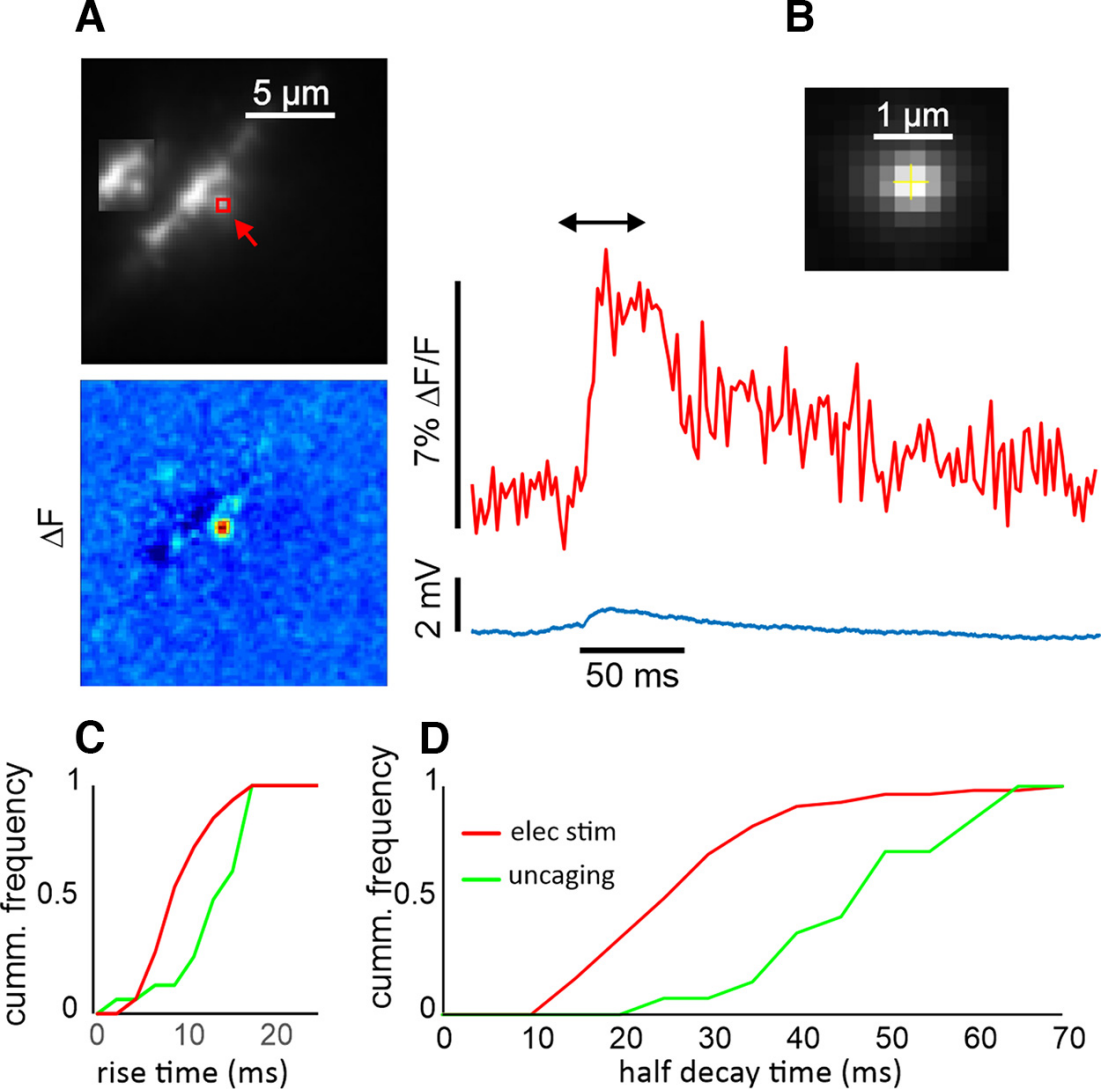
Time courses of Ca^2+^ transients evoked by uncaging MNI-glutamate on a dendritic spine detected with 150 μm OGB-5N. ***A***, Image of a dendritic segment with a targeted spine indicated with a red arrow. A 1-ms flash at 720 nm generated a small EPSP and a calcium transient from the red ROI. The image in the inset shows the same segment without the ROI to make the spine clearer. The pseudocolor difference image (between the times at the ends of the black arrow) shows that the [Ca^2+^]_i_ increase was only over the spine. ***B***, Enlargement of a part of an image of a fluorescent bead excited by an 800-nm two-photon flash. Each pixel is 0.2 μm. The FWHM of a profile through the image (data not shown) was 0.7 μm. ***C***, Cumulative frequency of rise times from many synaptically activated (*n* = 63) and uncaging (*n* = 16) calcium transients. ***D***, Similar cumulative frequencies of half decay times. There is a clear difference between the two profiles.

Figure 7*A* shows a typical experiment. We first confirmed that when properly aligned the pulsed laser activates a small spot. Figure 7*B* shows the fluorescence of a 4-μm bead detected at 500–600 nm when excited at 800 nm. The response, detected by the camera, had a FWHM of 0.7 μm). We then positioned a spine just next to the laser spot. In our experience, uncaging directly on the spine often generated fluorescence artifacts and bleached the indicator. Using a flash of 1.0 ms in duration, we generated a small uEPSP (1.1 ± 0.4 mV) and a calcium transient (Fig. 7*A*). The [Ca^2+^]_i_ change was confined to the region of the spine. The calcium signal had a fast rise time (∼10 ms) and a slower half decay time (∼50 ms). We repeated this experiment for many other spines, measuring their rise times and half decay times. For quantitative analysis we rejected signals that were not confined to identified spines, and which had strong stimulus artifacts. In some cases, the shape of the response was sensitive to the position of the ROI. For those responses we chose the location with the fastest kinetics. Figure 7*C*,*D* shows these times on a cumulative probability plot and compares them with similar times measured from synaptically activated responses (Fig. 4*B*). The rise times of synaptically evoked (7.9 ± 3.3 ms; *n* = 63) and uncaging evoked (10.8 ± 3.9 ms; *n* = 16) transients are close, although the *p*-value for a *t* test of the difference was 0.02. In contrast, as shown in Figure 7*D*, the half decay times were clearly different. The synaptically evoked half decay time (26.0 ± 11.5 ms; *n* = 55) was almost twice as fast as the uncaging evoked half decay time (49.4 ± 15.6 ms; *n* = 16). The *p*-value for the difference was 1.9 × 10^−5^. One issue of concern is that Figure 4*B* shows that the events with large EPSPs have the fastest decay times, which might be because of a strong contribution from VGCCs in addition to NMDA receptors. Even if we eliminate all events with EPSPs larger than 5 mV, the average half decay time for the synaptically activated events is 29.0 ± 14.3 ms (*n* = 26). This still differs from the uncaging half decay time with a *p*-value of 0.004. In summary, both synaptically evoked and uncaging evoked calcium transients are fast, but the half decay times of synaptically evoked transients are ∼25 ms faster.

## Discussion

### Calcium responses

These experiments extend previous measurements of calcium and sodium transients in dendritic spines. The new data result from the use of improved technology (Miyazaki et al., 2019) and the use of low-affinity indicators. Where comparable, our results using mice and the indicators SBFI and OGB-5N, were like those measured previously (Miyazaki and Ross, 2017) from rats using ING-2 and bis-fura-2. The most significant conclusions from the calcium experiments were that the rise time (∼7.7 ms) and the half decay time (∼26 ms for most EPSPs) of the synaptically activated calcium transients, were faster than found in many previous measurements (Kovalchuk et al., 2000; Sabatini et al., 2002; Nevian and Sakmann, 2006; Bloodgood and Sabatini, 2007; Enoki et al., 2009). These times are consistent with the finding that the contribution to the [Ca^2+^]_i_ increase by the EPSP driven relief of the Mg^2+^ block was the dominant component. Blockade of most of these signals by NBQX (Fig. 5) supports this conclusion. If Ca^2+^ entry through the NMDA receptor channels at resting potential was bigger both the rise time and half decay time would be slower, since these channels remain open for longer times than the EPSP. Since most of the Ca^2+^ entry is driven by the AMPA receptor mediated EPSP in the spine, it follows that processes that modulate AMPA receptor activation, like desensitization or potentiation, will have direct effects on Ca^2+^ signaling as well as affecting the synaptic potential.

One factor that influenced the generation of faster synaptic calcium signals in our experiments is that we used OGB-5N as the indicator. This indicator has a high K_d_ (∼40 μm), which makes it a weak buffer. Consequently, the time courses of the transients should be close to the responses expected without indicators in the cell. Previous experiments used relatively high concentrations of higher affinity indicators (OGB-1, fluo-5F, OGB-6F, fluo-4), which are stronger buffers. OGB-5N was used in some previous experiments (Noguchi et al., 2005), but those experiments were performed in ACSF with very low Mg^2+^, which generates much larger and slower NMDA calcium responses. We were able to make measurements with good S/N with this indicator, even with threshold synaptic stimulation and normal Mg^2+^ in the ACSF, because we used focal laser stimulation for fluorescence excitation and used a sensitive, high-speed CCD camera to detect the transients.

It is not obvious that calcium transients activated synaptically and transients activated by uncaging should have the same time course. Synaptically activated vesicular release of glutamate is expected to be confined to a spot ∼200 nm in diameter around the release site (Raghavachari and Lisman, 2004) and consequently is likely to rise and dissipate in 100 μs. In contrast, the two-photon uncaging spot covers a larger area (Zipfel et al., 2003; 700 nm in our experiments) and therefore will raise and dissipate the glutamate concentration more slowly than vesicular release does, even if the uncaging flash is a short as 0.5 ms, as in some experiments (Bloodgood et al., 2009). If AMPA receptors are more concentrated opposite the release site than the NMDA receptors, as suggested in some super resolution experiments (Choquet and Hosy, 2020), then electrical synaptic stimulation will preferentially target AMPA receptors. The consequences of this differential targeting are not clear since the receptors have different sensitivities to glutamate, react with different rate constants, and have different levels of desensitization. But the simplest conclusion is that many NMDA receptors, especially those away from the site of vesicular release, will be preferentially activated in two-photon uncaging experiments compared with activation by synaptic stimulation. This effect will be enhanced by the higher sensitivity of NMDA receptors to glutamate. This difference could explain the greater contribution of Ca^2+^ entry through NMDA receptors at resting potential (which persists longer than the AMPA-mediated current (Hestrin et al., 1990; Spruston et al., 1995a), in uncaging evoked signals compared with this component in synaptically evoked responses. Our experiments (Fig. 7), which found that the uncaging Ca^2+^ transients were ∼25 ms longer than electrically evoked transients, are consistent with this explanation. The importance of AMPA receptors in gating Ca^2+^ entry through NMDA receptors was discussed in previous experiments using different experimental approaches (Yuste et al., 1999; Bloodgood et al., 2009; Holbro et al., 2010; Chalifoux and Carter, 2011) but not quantitatively assessed. If most of the Ca^2+^ entry is gated by the brief AMPA-mediated EPSP in the spine, then it is reasonable that the decay time will be close to the decay time of [Ca^2+^]_i_ following a bAP since the EPSP in the spine is very fast (<8 ms; Popovic et al., 2015). The synaptically evoked decay time is slower because there is a small component of Ca^2+^ entry through NMDA receptor channels that persists after the spine EPSP returns to resting potential.

### Sodium responses

The results from the sodium measurements in mice support those previously reported from rats (Miyazaki and Ross, 2017). In more extensive measurements we continued to find that there was little evidence for voltage dependent Na^+^ entry in spines from either intrasomatically evoked or synaptically evoked bAPs. This conclusion agrees with one set of physiological experiments (Grunditz et al., 2008) and the lack of immunologic evidence for Na^+^ channels in spines (Lorincz and Nusser, 2010). However, it differs from several previous experiments. The reasons for this difference are not clear. Some possibilities are that we used electrical stimulation and other groups used glutamate uncaging (Araya et al., 2007; Bloodgood and Sabatini, 2007; Bywalez et al., 2015) or looked at spines from different kinds of neurons (Bywalez et al., 2015), or examined spines with long necks using long uncaging pulses (Araya et al., 2007). The spectrum of decay times of sodium transients was similar in both species (Fig. 2*C*) and similar with both sodium indicators (SBFI and ING-2), indicating that this spectrum reflects physiological or morphologic variation and not experimental error. These fast times for localized synaptic signals in spines but not from widespread signals from bAPs in dendrites (Rose et al., 1999) agree with the previous conclusion that the main removal process is diffusion of Na^+^ from spines into adjacent dendrites (Miyazaki and Ross, 2017). If diffusion times through spines is proportional to spine neck resistance, as suggested by the original analysis of Svoboda et al. (1996), then the rapid half decay times of these transients (∼21 ms), especially those at the faster end of the spectrum (Fig. 2*B*,*C*), are consistent with a low spine neck resistance (Svoboda et al., 1996; Popovic et al., 2015), although it is difficult to make a precise conversion of diffusion time into neck resistance (Tønnesen et al., 2014). Bloodgood and Sabatini (2005) also measured a spectrum of diffusion times in spines in organotypic hippocampal slice cultures using FRAP of small proteins (PGAFP) and small molecules (HPTS). These times were much longer than the times we measured for sodium diffusion. The source of this difference is not clear, although some is probably because of the difference in the size of the diffusing molecule.

Since there was little VGSC and/or NMDA receptor mediated Na^+^ entry, and only small VGCC Ca^2+^ entry, it follows that there was little regenerative component in the synaptic electrical response in the spine, casting doubt on theories that have proposed an important role for spine localized spikes in synaptic integration (Segev and Rall, 1988). However, it is clear that dendritic Na^+^ channels assist in the backpropagation of action potentials in dendrites (Stuart and Sakmann, 1994) and regenerative Na^+^ events can be evoked in the dendrites if stimulation is strong and focused (Golding and Spruston, 1998). Also, passive amplification of the spine EPSP can also occur when spine neck resistance is high (Harnett et al., 2012). Other experiments have demonstrated regenerative NMDA responses in spines following repetitive stimulation (Polsky et al., 2009), so our conclusions are restricted to conditions where only single synaptic responses are generated.

### Significance of fast AMPA receptor driven spine calcium kinetics

There are at least three important consequences to our findings of fast synaptic [Ca^2+^]_i_ changes. First, relates to the rate of activation of downstream processes in the spine. Molecules like calmodulin or CamKII may be activated to different extents if the [Ca^2+^]_i_ transients are briefer and larger than previous estimates. This timing window also might be relevant to spike timing-dependent plasticity (STDP), although we did not investigate this process. Another consequence is the potential generation of large [Ca^2+^]_i_ changes by repetitive synaptic activation. If the transients are very fast there will be little summation unless the synapses are activated within the duration time of single transients (∼40 ms). Slower transients, as suggested by experiments with higher affinity indicators, are more likely to summate. Third, is the question of whether uncaging evoked [Ca^2+^]_i_ changes are a good model for testing signaling mechanisms in the spine. If uncaging activates a different mixture of glutamate receptors with different kinetics than synaptic activation, the calcium dependent generation of signaling molecules like Ras may be different from previously proposed.

## Synthesis

Reviewing Editor: Alexey Semyanov, Shemyakin-Ovchinnikov Institute of Bioorganic Chemistry Decisions are customarily a result of the Reviewing Editor and the peer reviewers coming together and discussing their recommendations until a consensus is reached. When revisions are invited, a fact-based synthesis statement explaining their decision and outlining what is needed to prepare a revision will be listed below. The following reviewer(s) agreed to reveal their identity: Marco Canepari, Lawrence Cohen.

I am glad to receive positive evaluations of your manuscript from two reviewers, with which I agree entirely. The reviewers made a few minor comments and suggested to cited relevant references. Please see below.

Reviewer #1

The investigation of dendritic spines using 2-photon calcium imaging and glutamate uncaging has greatly advanced our understanding of postsynaptic processes, but the question of the physiological kinetics of glutamate receptors and associated calcium signals remained unresolved.

The detailed biophysical analysis reported in this study represents a significant progress in this direction. Specifically, it shows that Na+ enters spines mostly through AMPA receptors whereas Ca2+ enters mostly through NMDA receptors. However, this component elicited by glutamate uncaging was longer than that elicited by physiological synaptic stimulation. The consequence of this result poses the question on whether the commonly accepted approach of focal glutamate uncaging is actually a reliable model of postsynaptic activation in light of the different dynamics of calcium-dependent pathways and build-up of activity. Technically, this study was performed in the most possibly rigorous manner using several indicators with different affinities as control. Biophysically, the importance of the results goes far beyond the major result as it provides a quantitative measurement of the Ca2+ and Na+ dynamics in dendritic spines during physiological synaptic activity.

The manuscript, clearly organized and with all aspects critically discussed, should be published after addressing the points below.

1. The authors report a rise time of the Ca2+ signal (10-90%) associated with the bAP of 1.58{plus minus}0.14 ms (Fig.1), obtained by recording fluorescence from OG5N at 2 kHz. This value seems consistent with the accurate measurements of the Ca2+ current kinetics obtained in the same preparation (Jaafari et al., Biophys J 107:1280-8, 2014; Jaafari and Canepari, J Physiol 594:967-83, 2016) at 20 kHz. In these studies, where the indicator (OG5N) was the same although used at higher concentration and the temperature only ∼2 oC higher, the peak of the Ca2+ current (maximal slope of the rise time) was 400-600 μs delayed from the peak of the bAP. I think that these two studies should cited in support of the slower measurements reported here.

2. The very fast decay time of Na+ from the spines is due to diffusion, as clearly stated by the authors. Since diffusion depends on morphological constrains, I am wondering whether performing the same measurement at higher temporal resolution can reveal more precise information on the size of the spine neck. Indeed, the comparison with the Na+ measurement in the AIS (Fig.2A) clearly shows that when diffusion is negligible Na+ remains constant for ∼2ms, indicating that at this very short time scale the kinetics of the decay is entirely determined by diffusion from the spine to the dendritic bulk. But, on the same time, it seems that the acquisition rate is not enough to discriminate throughout a range of decay times.

3. If the Ca2+ signal localized to dendritic spines after NMDAR blockade is mediated by VGCC, then either the voltage transient in the dendritic spine is larger than that in the dendrite, contradicting other studies (Holthoff et al. J Physiol 588:1085-96, 2010; Popovic et al. 2015, already cited in the manuscript), or there is larger expression of (probably low-voltage activated) VGCCs in the spines. One should notice that the studies reporting a uniform membrane potential in dendrites and spines were actually performed in layer-5 pyramidal neurons, but in CA1 hippocampal pyramidal neurons the selective expression of functional R-type VGCC has been reported (Bloodgood and Sabatini, 2007, already cited in the manuscript). This is a very important issue that comes out from the results and therefore it should be addressed and discussed in extent.

Reviewer #2

This seems to be a careful set of experiments which establish an important result. The calcium and sodium changes in a spine in response to synaptic stimulation are very short-lived.

This seems to be a careful set of experiments which establish an important result. The calcium and sodium changes in a spine in response to synaptic stimulation are very short-lived.

Minor comments:

Could Na-Ca exchange remove sodium?

No mention of the spine neck resistance controversy? “Our results are consistent with e.g. Zecevic and Svoboda and not consistent with e.g. Magee and Yuste.”?

Figure suggestions:

Add titles for the Figures.

Add labels for stimulation types in Figure 1. Enlarge the images in figure 1A. Many figures would benefit from additional labels. Some fonts are too small.

There are no significance indicators in Figure 6B.

The authors are not well hidden. For example, “For simultaneous sodium and calcium imaging we used the apparatus previously described (Miyazaki and Ross, 2015; Miyazaki et al., 2019).” And, there were 11 references to Miyazaki and Ross, 2017.

## Author Response

We thank the reviewers for their comments which were mostly positive. As the editor noted, the requested changes were minor. We address them below.

Reviewer 1:

1) The reviewer suggests that we add a reference to other work that measured the rise time of calcium signals evoked by action potentials. We added a reference to Jaafari et al. (2014). We thought one reference was sufficient for this point.

2) The reviewer suggests that measurements at higher time resolution might reveal some additional information. We agree. However, these measurements are at the limit of our current apparatus. Possibly, future experiments will explore this opportunity.

3) The reviewer suggests that the synaptically activated calcium signal in APV, which should be due to activation of VGCCs, might reveal something about the distribution of VGCCs in the spine and dendrite and/or the comparative EPSP amplitude in the spine and nearby dendrite. However, his suggestion depends on the confinement of this signal to the spine. We did not make this claim.

Reviewer 2:

1) The reviewer asks if Na-Ca exchange could remove sodium. We did not test this possibility. However, Rose et al. (1999) argued that the temperature sensitivity of sodium removal when there is no diffusion, indicates that a pump is the ultimate removal mechanism. Even if an exchanger is involved it is too slow to be responsible for the fast removal from spines.

2) We chose not to make a strong statement about spine neck resistance. We feel that some parameters used in the conversion of sodium decay time to spine neck resistance on not known well enough to make a conclusion from our data. However, we did add references to Popovic et al. (2015) AND Svoboda et al. (1996), which relate to the controversy.

3) The figures have titles. The eNeuro format does not bold them.

4) We modified some of the figures and increased the font size in the figure text as suggested.

5) We removed the significance bars in Fig. 6B. Instead, the p values for difference in values are indicated in the figure legend.

6) We did not intend to hide the authors. We indicated to the editor that we did not want the reviews to be double blind. Apparently, this point was not communicated to the reviewer.
