# Supplementary figures and images for: Fast Synaptically Activated Calcium and Sodium Kinetics in Hippocampal Pyramidal Neuron Dendritic Spines
Source: eNeuro. 2022 Nov 25;9(6):ENEURO.0396-22.2022. doi: 10.1523/ENEURO.0396-22.2022 (PMC9718353; doi:10.1523/ENEURO.0396-22.2022)

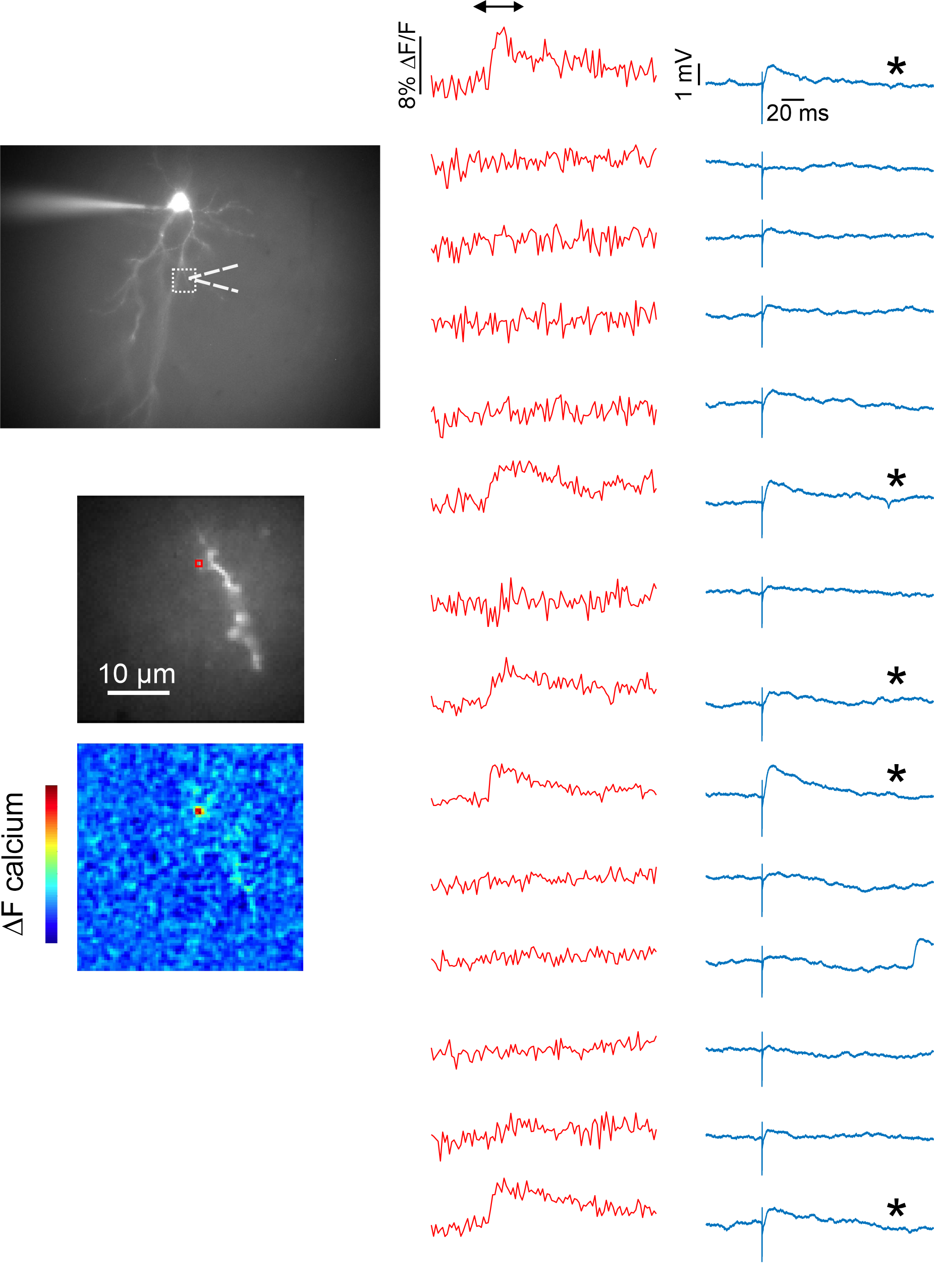

Supplement: Extended Data Figure 4-1 — An example of a spine where synaptic calcium signals were probably evoked by activating a single presynaptic axon. The top image shows a low magnification picture of the pyramidal neuron filled with fluorescent indicator. The position to the stimulating electrode is shown. The dotted box indicates the region examined at higher magnification in the image below. The ROI is indicated with a small red box. The difference image of the evoked Ca2+ signal (as in Fig. 1) is shown below. The traces on the right show 14 calcium signals and somatic electrical recordings in response to single weak electrical shocks. Five traces, marked with an asterisk, have both electrical and optical responses; the others did not respond (see also Enoki et al., 2009) Using this assay, we concluded that only a single presynaptic fiber was activated, with stochastic success in releasing transmitter. For these five active responses the average EPSP amplitude was 0.85 ± 0.24 mV; the average calcium rise time was 11.4 ± 2.5 ms, and the average half decay time was 31.4 ± 10.9 ms. Download Figure 4-1, file. [file enu-eN-NWR-0396-22-s02.tif]
